# Transcriptomic Meta-Analysis Unveils Shared Neurodevelopmental Toxicity Pathways and Sex-Specific Transcriptional Signatures of Established Neurotoxicants and Polystyrene Nanoplastics as an Emerging Contaminant

**DOI:** 10.3390/toxics13080613

**Published:** 2025-07-22

**Authors:** Wenhao Wang, Yutong Liu, Nanxin Ma, Rui Wang, Lifan Fan, Chen Chen, Qiqi Yan, Zhihua Ren, Xia Ning, Shuting Wei, Tingting Ku

**Affiliations:** 1 Shanxi Key Laboratory of Coal-Based Emerging Pollutant Identification and Risk Control, Research Center of Environment and Health, College of Environment and Resource, Shanxi University, Taiyuan 030006, China; wwh11232002@163.com (W.W.); 202313905002@email.sxu.edu.cn (Y.L.); mananxin2016@163.com (N.M.); wir010202@163.com (R.W.); 202223905001@email.sxu.edu.cn (L.F.); 13347541630@163.com (C.C.); yqq13134680242@163.com (Q.Y.); zhren@sxu.edu.cn (Z.R.); ningxia@sxu.edu.cn (X.N.); 2 First Hospital/First Clinical Medical College, Shanxi Medical University, Taiyuan 030001, China; 3 NHC Key Laboratory of Pneumoconiosis, Taiyuan 030001, China

**Keywords:** neurodevelopmental toxicity, transcriptome meta-analysis, legacy neurotoxicants, polystyrene nanoplastics

## Abstract

Environmental contaminants exhibit heterogeneous neurotoxicity profiles, yet systematic comparisons between legacy neurotoxicants and emerging pollutants remain scarce. To address this gap, we implemented an integrative transcriptome meta-analysis framework that harmonized eight transcriptomic datasets spanning in vivo and in vitro neural models exposed to two legacy neurotoxicants (bisphenol A [BPA], 2, 2′, 4, 4′-tetrabromodiphenyl ether [BDE-47]) and polystyrene nanoplastics (PSNPs) as an emerging contaminant. Our analysis revealed a substantial overlap (68% consistency) in differentially expressed genes (DEGs) between BPA and PSNPs, with shared enrichment in extracellular matrix disruption pathways (e.g., “fibronectin binding” and “collagen binding”, *p* < 0.05). Network-based toxicogenomic mapping linked all three contaminants to six neurological disorders, with BPA showing the strongest associations with Hepatolenticular Degeneration. Crucially, a sex-stratified analysis uncovered male-specific transcriptional responses to BPA (e.g., lipid metabolism and immune response dysregulation), whereas female models showed no equivalent enrichment. This highlights the sex-specific transcriptional characteristics of BPA exposure. This study establishes a novel computational toxicology workflow that bridges legacy and emerging contaminant research, providing mechanistic insights for chemical prioritization and gender-specific risk assessment.

## 1. Introduction

With the continuous innovation and large-scale manufacturing of industrial products, an increasing number of environmental contaminants (ECs) have been generated. Despite often being present at low environmental concentrations, they exhibit widespread bio-toxicity, environmental persistence, and a potential for bioaccumulation [1]. These factors could pose significant adverse effects on human health and ecosystems [2,3]. Therefore, it is imperative to evaluate the potential risks of ECs to human health.

Neurodevelopmental disorders impact millions of individuals globally, with a significant rise in the diagnoses of autism spectrum disorder (ASD) and attention-deficit/hyperactivity disorder (ADHD) [4]. ECs include many synthetic or natural substances, such as pharmaceuticals, personal care products, hormones, flame retardants, and endocrine disruptors [5,6], which are not often controlled or monitored in the environment and are widely disseminated through industrial emissions, making them major contributors to neurodevelopmental toxicity [7]. Even low-level exposure to certain ECs has the potential to impair brain function and disrupt neurodevelopment [8,9]. Thus, assessing the risks of these ECs to neurodevelopment is of paramount importance.

Among the known ECs, two legacy neurotoxicants—bisphenol A (BPA) and 2, 2′, 4, 4′-tetrabromodiphenyl ether (BDE-47)—alongside polystyrene nanoplastics (PSNPs) as an emerging contaminant, are of particular concern. BPA, as one of the most extensively produced synthetic chemicals worldwide, is commonly found in epoxy resins and polycarbonate plastics [10]. It has been reported that BPA exposure can disrupt multiple brain structures and induce molecular alterations, including the impairment of the blood–brain barrier and changes in the expression levels of several key genes and proteins [11]. Notably, nanopollutants at the nanoscale can pass through the umbilical cord to reach the fetus and can cross the placental barrier [12,13], thereby posing health risks to both the mother and the offspring. Even low-dose exposure during pregnancy has been linked to the development of behavioral disorders in offspring [14,15]. Similarly, BDE-47, a commonly used flame retardant, is widely present in textiles, furniture, and electronic products [16]. It has been shown to interfere with cholinergic receptor responses during prenatal exposure, which is closely associated with behavior and cognitive functions [17]. In addition, as a typical nanomaterial, PSNPs are used as the model contaminant in over 70% of laboratory toxicology studies. Their concentrations in the environment have been reported to reach reach μg/L levels [18], potentially posing significant risks to human health. Studies using zebrafish as a model organism have demonstrated that PSNPs induce neurotoxicity through their effects on neurotransmission and neurodevelopment [19]. Notably, these pollutants may share the same mechanism when inducing neurotoxicity in organisms. For example, all three pollutants may induce neurotoxicity through the pathway of ferroptosis [20,21,22], a programmed cell death pathway driven by iron-dependent lipid peroxidation. However, due to the complex interplay of synergistic effects from multiple pollutants and the limitations of traditional toxicological methods, elucidating the common hazard mechanisms underlying multi-pollutant exposures remains a significant scientific challenge. Currently, the rapid accumulation of open-access biological data and advancements in bioinformatics provide unprecedented opportunities to address these complexities [23]. For example, researchers have used bioinformatics approaches to identify certain ferroptosis-related genes as biomarkers for specific diseases [24]. Specifically, computational strategies integrating multi-transcriptome meta-analysis enable a systematic decoding of toxicological interactions, molecular pathways, and disease associations across heterogeneous contaminants. Capitalizing on these developments, we implemented a novel bioinformatics pipeline to comparatively dissect the neurotoxic mechanisms of three structurally distinct ECs.

In this study, neurotoxicity-related omics data for BPA, BDE-47, and PSNPs were systematically retrieved from the NCBI GEO database, encompassing both microarray and RNA-seq platforms. To ensure analytical robustness, we (1) identified the DEGs of the three pollutants and used gene co-expression network mining to uncover their conserved neurotoxic pathways, (2) used the curated neurological disease database to perform an association analysis with the data of the DEGs, mapping the relationship between pollutants and diseases, and (3) conducted an in-depth analysis of specific databases to reveal sex-specific responses. Through a meta-analysis of eight independent datasets, we not only revealed divergent neurotoxic effects between legacy and emerging contaminants but also identified sex-dimorphic transcriptional responses specific to BPA exposure. These findings provide a mechanistic basis for understanding shared toxicity pathways (e.g., extracellular matrix dysregulation) and pollutant-specific disease associations, while highlighting the necessity of gender-informed risk assessment frameworks.

## 2. Materials and Methods

### 2.1. Identification and Organization of Datasets

We retrieved transcriptomic data related to the three ECs under investigation from the GEO DataSets of the NCBI (National Center for Biotechnology Information). In the search process, both the full names and abbreviations of the ECs were considered to ensure no datasets were overlooked. For Bisphenol A, search terms included “Bisphenol A”, “4, 4′-propane-2,2-diyldiphenol”, and “BPA”. For BDE-47, the search terms included “BDE-47” and “2, 2′, 4, 4′-Tetrabromodiphenyl ether”. For polystyrene microplastics, the search terms included “Polystyrene microspheres”, “Polystyrene nanoparticles”, “PS nanoparticles”, “PSNPs”, and “PSMPs”.

**Figure 1:**
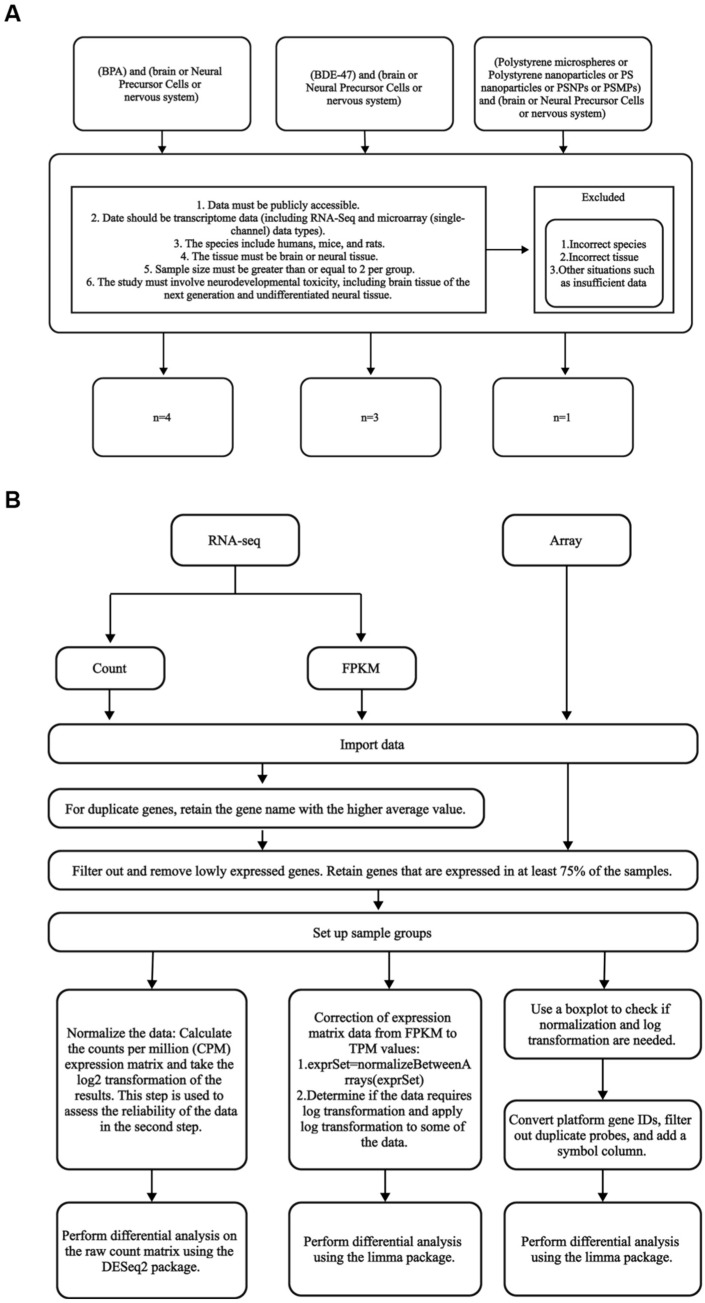
Overall data curation workflow and analysis pipeline. (**A**) Criteria and selection workflow of GEO transcriptome datasets included in the study. (**B**) Meta-analysis pipeline for both RNA-seq and array data types. Reproduced from [25]. © 2023 The Authors. Published by Elsevier Ltd.

Subsequently, we screened the datasets based on the following criteria (Figure 1A): (1) data must be publicly accessible; (2) data should be transcriptome data (including RNA-Seq and microarray (single-channel) data types); (3) the inclusion criteria should encompass humans, mice, and rats; (4) the tissue must be brain or neural tissue; (5) the sample size must be greater than or equal to 2 per group; and (6) the study must involve neurodevelopmental toxicity, including brain tissue of the next generation and undifferentiated neural tissue. All GEO transcriptomic profiles accessible at the end of July 2024 were considered.

### 2.2. Downloading and Processing Transcriptomic Data Profiles

We used the R package GEOquery (version: 4.3.2) to directly download transcriptomic datasets from GEO, including data types such as count data, FPKM data, microarray data, and their corresponding annotation platforms. The downloaded data were then imported into RStudio (version 4.3.2) for further processing. The count data were cleaned using the na.omit function to remove genes containing missing values. In the case of duplicate genes, we retained the gene expression data with the highest average expression value to ensure each gene appeared only once and represented its major expression profile across all samples. The data were filtered to retain genes that were expressed in at least 75% of the samples, ensuring that the retained genes had sufficient expression information in most of the samples. The data were then standardized and normalized. We converted FPKM data to TPM values, followed by log2 transformation (log2 (exprSet + 1)) to make the data distribution closer to a normal distribution, reduce the impact of extreme values, and facilitate subsequent analysis. The normalizeBetweenArrays function from the limma package (version 3.58.1) was applied to normalize the expression matrix, effectively removing technical differences between samples. Finally, for microarray data, appropriate log transformation and normalization were also performed to ensure data quality and comparability (Figure 1B).

To ensure the consistency and comparability of subsequent cross-species pathway enrichment analysis results, the homologene function was used to establish effective mapping between the gene symbols of mice (Mus musculus), rats (Rattus norvegicus), and humans, based on orthologous gene relationships. Gene symbols from the Human Gene Nomenclature Committee (HGNC) were used to ensure the accurate matching of mouse, rat, and human genes.

### 2.3. Transcriptional Characteristics Clustering and Differential Gene Expression Analysis

The gene expression data from multiple datasets of the same pollutant are subjected to a correlation analysis to identify and visualize the gene expression patterns across different datasets. This approach evaluates the consistency of gene expression changes across datasets related to the same pollutant, allowing for the selection of representative datasets. For studies involving multiple dosages, each dosage is treated as an independent dataset to identify dosage-specific gene expression characteristics. Before performing the analysis, data preprocessing and outlier detection are carried out. The absolute median deviation (MAD) method is used to select the top 500 genes with the highest variability, minimizing the impact of low-expression or low-variability genes on the results. This ensures the statistical significance and reliability of the analysis. Subsequently, the cor function in R is applied, using the Spearman rank correlation method, to analyze the correlation of fold-change (logFC) values between the treatment and control groups for all genes. This helps reveal common patterns of gene changes across different samples. A higher positive correlation coefficient indicates stronger consistency in gene expression changes across datasets, while a negative correlation coefficient suggests opposing trends. Finally, the corrplot package is used to visualize the correlation matrix, which is annotated with sample information. A correlation heatmap is generated using the pheatmap package to visualize the gene expression relationships, laying the foundation for subsequent differential expression analysis.

For differential gene expression analysis, the limma package is used for FPKM and microarray data. Based on a linear model, limma compares the expression differences between experimental and control groups through appropriate modeling and empirical Bayes estimation, identifying DEGs with statistical significance. For raw count data, the DESeq2 package (version 1.42.1) is more appropriate. DESeq2 models gene expression using a negative binomial distribution and normalizes the data, evaluating the differences between conditions (e.g., experimental versus control groups). It effectively controls for biases due to factors like sequencing depth and gene length, minimizing false positives. When selecting DEGs, genes with a *p*-value < 0.05 (indicating statistical significance) and |log2 fold change| > 0.585 (indicating a fold change greater than 1.5) are considered as DEGs. These criteria help identify biologically significant gene changes while avoiding false positives caused by small changes. This approach allows for the systematic identification of DEGs with biological relevance under experimental conditions, providing a basis for subsequent functional enrichment analysis and biological research.

### 2.4. Gene Set Enrichment Analysis (GSEA), Pathway, and Disease Gene Enrichment Analysis

To identify the overall gene enrichment differences between the exposed and control groups, Gene Set Enrichment Analysis (GSEA) is conducted using the clusterProfiler package (version 4.10.1) in R. In brief, the gene expression matrix and corresponding fold-change values are imported and ranked in descending order. Then, the gseGO function is used for analysis, with the org.Hs.eg.db database and default parameters (nPerm = 1000, minGSSize = 10, maxGSSize = 1000, and pvalueCutoff = 0.05). Enrichment plots for the top 5 Gene Ontology (GO) terms are generated using the gseaplot2 function for each group.

A further functional enrichment analysis is performed on the intersection of differentially expressed genes (DEGs) using the DAVID database. The species is set to Homo sapiens, and the data type is selected accordingly. A GO enrichment analysis is performed to investigate the biological processes (BP), cellular components (CC), and molecular functions (MF) of the predicted DEGs. The KEGG database in DAVID is used to predict and visualize the pathways involved in the DEGs. Only terms with a *p*-value < 0.05 are considered statistically significant and enriched. Additionally, the enrichment terms, *p*-values, and counts are input into a bioinformatics platform to generate visualizations such as GO enrichment bubble plots and KEGG enrichment bar charts. These visualizations help present the enrichment results in a clear and interpretable manner.

### 2.5. Weighted Key Driver Analysis (wKDA) of DEGs

To identify the potential gene regulatory networks and key drivers (KDs) associated with the three ECs, a Weighted Key Driver Analysis (wKDA) is performed using Mergeomics and its constructed pollutant-specific Bayesian brain networks. The upregulated and downregulated DEGs of each pollutant cluster are separately used for wKDA analysis. The key drivers (KDs) of each DEG set and their corresponding subnetworks are visualized. These subnetworks are enriched with cluster-specific upregulated or downregulated DEGs, with an FDR < 0.05. Genes identified within these subnetworks are considered significant key drivers (KDs).

### 2.6. DEG-Disease Association Analysis

The association between the differentially expressed genes (DEGs) and diseases is investigated using the CTD database, by calculating the Jaccard similarity coefficient. A correlation heatmap is generated to visualize these associations. The Jaccard similarity coefficient is a commonly used metric to measure the similarity between two sets. It is defined as the size of the intersection of the two sets divided by the size of their union. The formula is as follows:

$$J\left(A,B\right)=\frac{\left|A\cap B\right|}{\left|A\cup B\right|}$$

$J\left(A,B\right)$: The Jaccard similarity coefficient between sets *A* and *B*, which measures the similarity of the two sets. It ranges from 0 to 1. *A* value of 0 indicates that the two sets are completely disjoint, while a value of 1 indicates that the two sets are identical.

$\left|A\cap B\right|$: The cardinality (number of elements) of the intersection of sets *A* and *B*. It represents the number of elements that are common to both set *A* and set *B*.

$\left|A\cup B\right|$: The cardinality of the union of sets *A* and *B*. It represents the total number of distinct elements in either set *A* or set *B* (or both).

## 3. Results and Discussion

### 3.1. A Series of Criteria Are Used to Filter the Dataset Under Study

**Figure 2:**
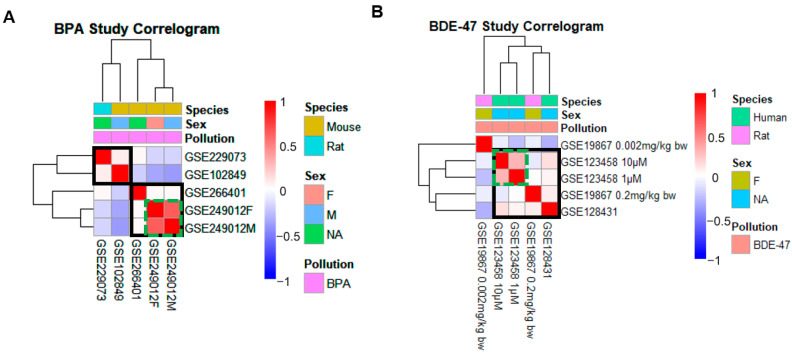
Cluster analysis for each EC based on similarity in gene signatures defines main EC study clusters. Correlograms were generated via the hierarchical clustering of gene signatures of ECs based on Spearman’s rank correlation coefficients of transcriptome response (logFC) between treatment and control groups for BPA (**A**) and BDE-47 (**B**). Studies were labeled using the GSE accession number, gender, and dose. Color of the heat map indicates Spearman’s rank correlation coefficients where red tones represent positive correlations and blue tones represent negative correlations. Solid black lines represent identified EC clusters used for downstream analysis. Green dashed lines highlight the groups with better correlation. The factors such as pollution, species, and sex are shown above the cluster map. Reproduced from [25]. © 2023 The Authors. Published by Elsevier Ltd.

A total of eight transcriptome datasets passed our selection criteria for inclusion (Table 1), including four for BPA, three for BDE-47, and one for PSNPs. The eight datasets include human, mice, and rat models. To determine the similarities and differences in gene responses to ECs exposure across studies, Spearman correlations of the logFC of genes between the treatment and control groups of each study were calculated against the logFC values from other studies to assess similarity in gene expression changing patterns. The results revealed that for BPA, the GSE249012 dataset exhibited similar gene expression across different sexes (Figure 2A). Consequently, we did not distinguish by sex and treated the dataset as a unified group. In the analysis of BDE-47 (Figure 2B), the GSE123458 dataset demonstrated consistent changes across various concentrations, and we selected the higher concentration (10 μM) for further investigation. Furthermore, the low concentration group in the GSE19867 dataset showed a poor correlation with other datasets, prompting its exclusion from subsequent analyses. Thus, the datasets selected for differential analysis include the following: the BPA-treated datasets (GSE229073, GSE266401, GSE249012, GSE102849); the BDE-47-treated datasets (GSE123458, GSE19867, GSE128431); and the PSNPs-treated dataset (GSE243612).

Species are abbreviated as follows: human (Hs), rat (Rn), and mouse (Mm). Routes of exposure are abbreviated as follows: in vitro (IV), oral gavage (OG), diet (DD-C for chemicals added in the chow), tail injection (TI), and subcutaneous injection (SI). Italicized values in the dose per body weight (Dose/bw) column indicate in vitro studies for which no dose/bw are available. Sexes are abbreviated as male (M) and female (F).

### 3.2. Biological Processes Affected by the Three ECs Differ Significantly

**Figure 3:**
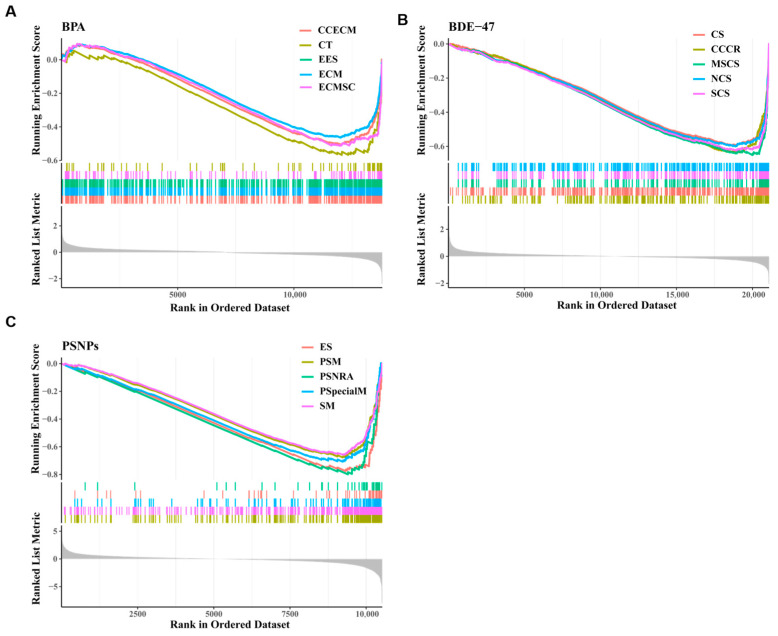
GSEA between the exposure and the control group in BPA (**A**), BDE-47 (**B**), and PSNPs (**C**). Top 5 gene sets were shown: Collagen-containing extracellular matrix (CCECM), extracellular matrix (ECM), external encapsulating structure (EES), extracellular matrix structural constituent (ECMSC), collagen trimer (CT), mitotic sister chromatid segregation (MSCS), chromosome segregation (CS), sister chromatid separation (SCS), nuclear chromosome segregation (NCS), condensed chromosome and centromere region (CCCR), postsynaptic membrane (PSM), synaptic membrane (SM), postsynaptic specialization membrane (PSpecialM), excitatory synapses (ES), and postsynaptic neurotransmitter receptor activity (PSNRA).

A Gene Set Enrichment Analysis (GSEA) was performed on the genes from the selected samples, and the results indicated that genes treated with BPA were significantly enriched in collagen-containing extracellular matrix, extracellular matrix, external encapsulating structure, extracellular matrix structural constituent, and collagen trimer. These five pathways are related to the function of the extracellular matrix and its components, which can regulate key processes in the development, activity, and growth of neurons (Figure 3A). This suggests that BPA exposure may impact the structural stability of neuronal processes and synaptic connections during the maturation of the central nervous system [26]. For BDE-47 treatment (Figure 3B), genes were significantly enriched in mitotic sister chromatid segregation, chromosome segregation, sister chromatid separation, nuclear chromosome segregation, condensed chromosome, and centromere region, which are regulatory pathways related to mitosis. However, abnormal mitosis may be a direct factor triggering brain injury [27]. Recent studies have shown that synapse dysfunction and loss are hallmarks of neurodegenerative diseases that correlate with cognitive decline [28,29]. The treatment with PSNPs significantly enriched genes associated with synapse-related pathways (Figure 3C), such as regulation of the postsynaptic membrane, synaptic membrane, postsynaptic specialization membrane, excitatory synapses, and postsynaptic neurotransmitter receptor activity, suggesting that PSNPs may have a significant effect on synaptic structures. In conclusion, the GSEA analysis indicates that the three ECs have significantly different focal points in their effects on neurodevelopment, suggesting divergent molecular pathways and distinct mechanistic targets may underlie their neurotoxicological profiles.

### 3.3. Similar Mechanisms of Neurotoxic Effects of BPA and PSNPs Compared to BDE-47 Are Identified

**Figure 4:**
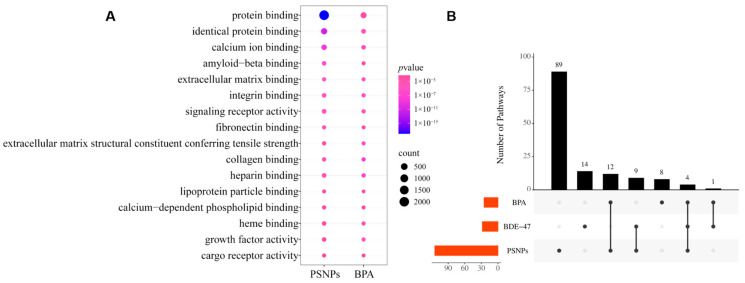
Comparison of genes across different EC clusters. (**A**) The heatmap shows GO terms associated with DEGs, with color coding based on the enrichment level of genes. The color variation of the circles indicates the significance of enrichment, and the size of the circles represents the gene count. The specific *p* values can be found in the figure legend. (**B**) The upset plot of significant KEGG pathways (FDR < 0.05) shows the enrichment of differentially expressed genes. The horizontal bars (set size) represent the total number of pathways for each cluster in each plot. In the upset plot, the dots point to specific EC clusters, where vertical bars show the pathway counts, and vertical lines between dots represent the intersections between two or more clusters.

To further investigate the potential similarities and differences in the mechanisms of action of the three ECs, we performed GO and KEGG analyses on all DEGs from the gene datasets of the three EC types to identify their conserved molecular functions, biological processes, and pathways. While no common genes were found in the shared GO terms across all three ECs (Figure 4A), we observed a large number of shared genes between PSNPs and BPA, with more than 2000 common genes in the protein binding category. Specifically, the shared processes between PSNPs and BPA may reflect their roles in regulating intracellular and extracellular signaling, maintaining cytoskeletal stability, and potentially inducing pro-inflammatory responses. Enrichment in processes such as “fibronectin binding” and “collagen binding” particularly suggests that these two pollutants might influence the structure and function of the extracellular matrix, thereby disrupting cell environment interactions. However, as a persistent organic pollutant, BDE-47 may impact cellular functions through different molecular targets or more specific pathways. For example, it can impact embryo development by inducing mitochondrial dysfunction or endoplasmic reticulum stress [30]. In a subsequent KEGG analysis, we successfully identified common pathways shared by the three ECs (Figure 4B), including the MAPK signaling pathway, cancer pathway, Rap1 signaling pathway, and PI3K-Akt signaling pathway. These pathways play crucial roles in cell proliferation, survival, differentiation, and stress responses, and are closely linked to the onset and progression of various diseases [31,32]. We found that the three pollutants shared partial pathway convergence, suggesting that they may have shared regulatory elements in inducing neurotoxicity. This could provide a basis for exploring their common neurotoxic mechanisms.

**Figure 5:**
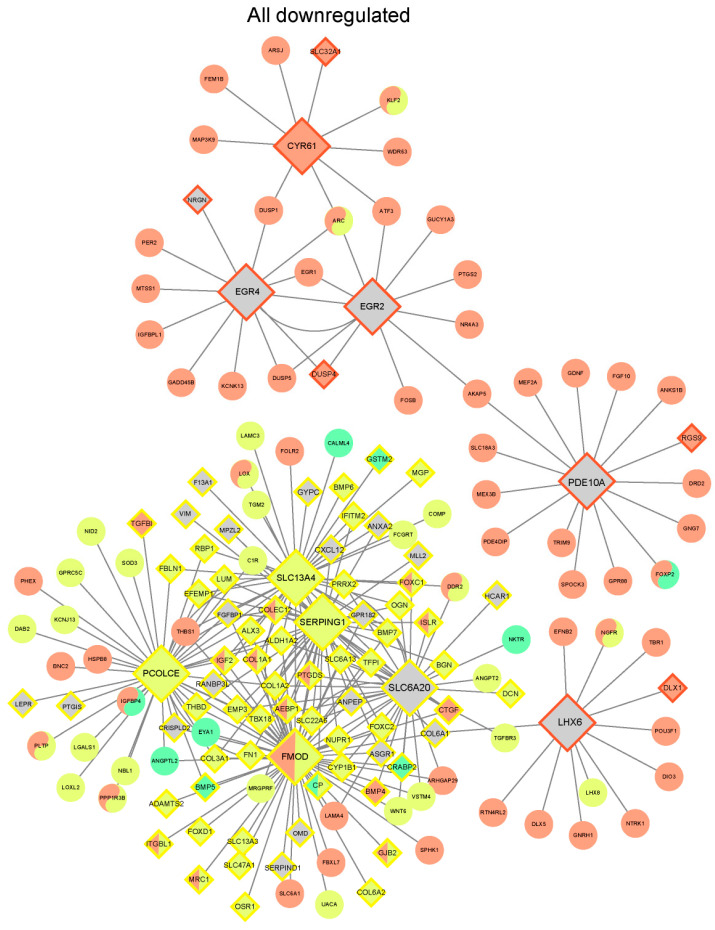
Network modeling identifies the overlapping KDs of three EC clusters and their subnetworks. All downregulated genes. Circles represent ordinary nodes of the Bayesian subnetwork, while boxes represent key driver nodes (KDs). The border color of the KDs indicates whether the node is a key driver for a particular module, with the color representing the associated module. Red, yellow, and green represent the DEGs of PSNPs, BPA, and BDE-47 under three different treatment conditions, respectively. Gray indicates genes in the network that are not DEGs of the aforementioned three pollutants.

Subsequently, we focused on exploring the differential genes of the three pollutants. We associated their upregulated and downregulated differential genes with the Bayesian brain network and identified a total of 21 Key Driver Nodes (KDs) for PSNPs (all downregulated) and 73 KDs for BPA (all downregulated). Although there were no shared KDs among the clusters of all three pollutants, there was a significant overlap between the downregulated gene clusters of PSNPs and BPA (Figure 5), including 11 overlapping KDs, such as AEBP1, COL1A1, COLEC12, and others. Among these, AEBP1 may be an oncogene and could serve as a new effective therapeutic target for glioma [33]. Furthermore, the interaction between AEBP1 and COLEC12 might damage the cerebral cortex, potentially contributing to the development of Alzheimer’s disease. Both could serve as valuable biomarkers for monitoring and treating neurological diseases [34]. Overall, these findings are consistent with what we observed at the pathway level. The regulatory mechanisms of BPA and PSNPs show some similarities, while the BDE-47 cluster exhibits KDs that are highly distinct from those of the other two clusters.

### 3.4. DEGs of EC Clusters Are Enriched for Diverse Pathways

**Figure 6:**
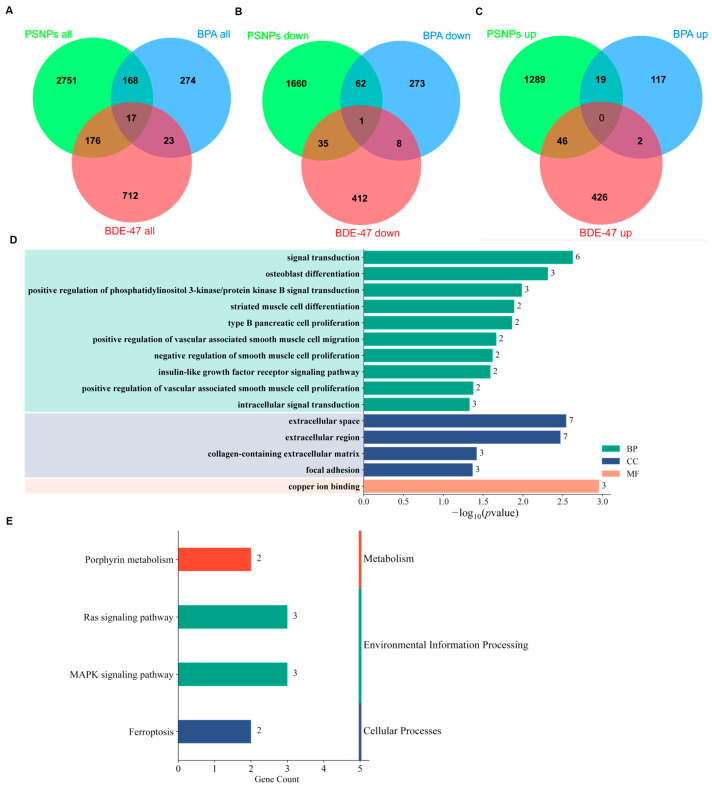
Differentially expressed genes (DEGs) overlap analysis and enrichment analysis. (**A**) The intersections of DEGs from the three ECs. (**B**) The intersections of all downregulated DEGs from the three ECs. (**C**) The intersections of all upregulated DEGs from the three ECs. (**D**) GO enrichment analysis of the 17 common DEGs across the three ECs. The green, blue, and orange bars represent Biological Process (BP), Cell Component (CC), and Molecular Function (MF) entries, respectively (*p* < 0.05). (**E**) KEGG enrichment analysis of the 17 common DEGs across the three ECs.

We employed a novel systematic analysis approach to handle different datasets, which effectively overcame the heterogeneity of the study and provided reliable information on EC-induced DEGs, pathways, or networks. The DEG results obtained from the three ECs revealed that PSNPs had the highest number of differentially expressed genes, totaling 3112, while BPA had the fewest, with only 482. Through an overlap analysis of the DEG results from the three ECs, we identified 17 overlapping genes (Figure 6A–C). These include AKAP12, which is associated with blood–brain barrier function and integrity [35], and PDE7B, which is linked to schizophrenia [36], among others. To further explore the functions of these 17 overlapping genes, we performed a GO enrichment analysis (Figure 6D). The results showed that six of these genes are involved in cell signaling transduction, suggesting that pollutants may interfere with cell proliferation, survival, and metabolism processes by regulating certain key signaling pathways, such as the PI3K/Akt signaling pathway and the insulin-like growth factor receptor signaling pathway. In terms of cellular components, genes exposed to pollutants were significantly enriched in the extracellular space and extracellular region, indicating that pollutants may disrupt the stability of the brain microenvironment by influencing the extracellular matrix and cell-to-cell interactions, ultimately affecting cell signaling and the maintenance of neural function. Notably, genes enriched in pathways related to the collagen-containing extracellular matrix and focal adhesion imply that pollutants may interfere with the formation and repair of neural networks by disrupting cell attachment, migration, and morphological remodeling. Building on the previous findings related to cellular components, in terms of molecular functions, three genes are associated with copper ion binding, indicating that pollutants may disrupt metal ion metabolism, thereby impacting the health and function of the nervous system. A further KEGG analysis revealed that these genes regulate common signaling pathways (Figure 6E), including porphyrin metabolism, Ras signaling, MAPK signaling, and ferroptosis. These pathways are critical for neural development, and their disruption could lead to a variety of neurodevelopmental disorders [37,38].

### 3.5. Gene-Disease Association Analysis Focuses on Some Key Neurological Diseases

**Figure 7:**
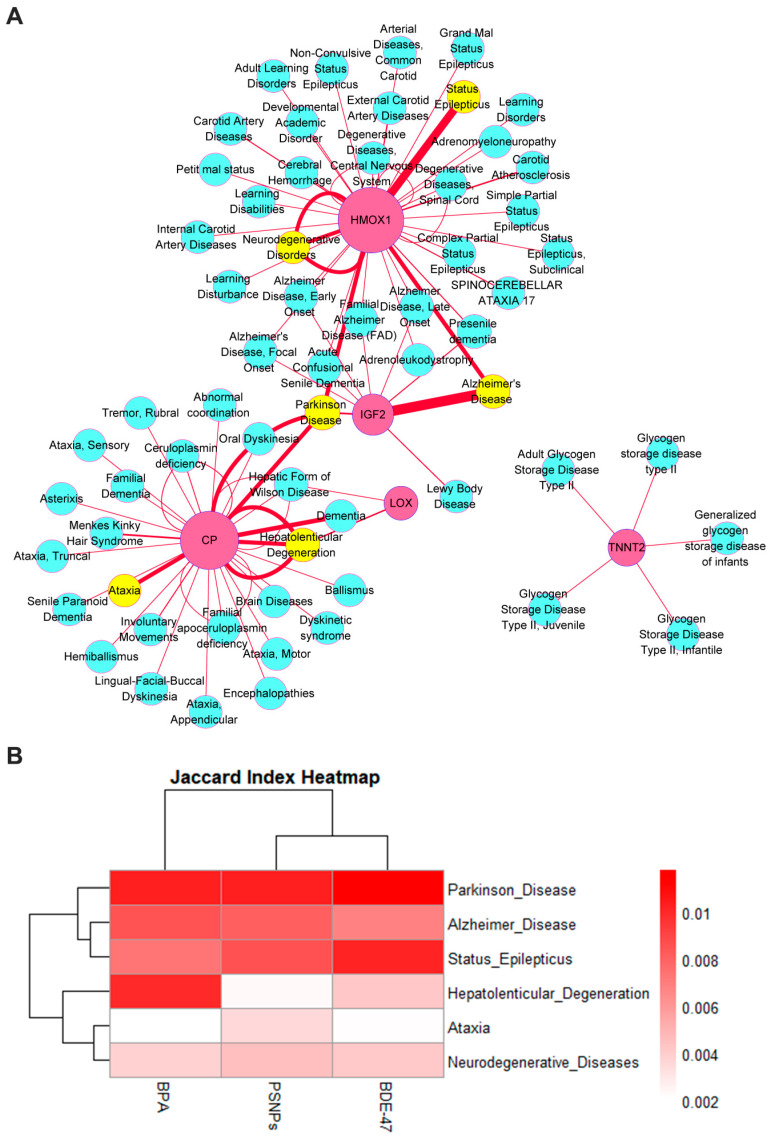
Disease association analysis of the 17 common DEGs. (**A**) The network diagram visualizes the association between genes and related neurological diseases. In the network diagram, the pink circles represent the common DEGs of the three ECs, while the blue circles indicate the neurological diseases in the CTD database that are known to be associated with these genes. The size of the nodes is positively correlated with the degree (i.e., the number of edges connected to the node), and the width of the connecting lines reflects the strength of the association between the genes and diseases. The stronger the association, the thicker the connecting line. Neurological diseases that are significantly associated with these genes are marked in yellow based on the width of the connecting lines. (**B**) The heatmap visualizes the correlation between 6 neurological diseases and the three ECs. The color of the heatmap represents the magnitude of the similarity coefficient, with a gradient from “white” to “red”. The darker the color, the higher the similarity.

Previous studies have suggested that gene expression and regulation can be highly tissue-specific, and most disease-related genes have tissue-specific expression abnormalities [39], which suggests that certain genes could function as biomarkers for predicting the development of specific diseases. In recent years, with the advancement of microarray and high throughput technology, bioinformatics methods have been increasingly employed in disease diagnosis, targeted therapy, and the prediction of biomarkers [40]. Many scientists have applied methods of transcriptome meta-analysis to identify biomarkers for various diseases, including CDKN1B and TFAM for Non-alcoholic fatty liver disease [40,41], INHBA, FNBP1, PDE9A, HIST1H2BG, and CADM3 for colorectal cancer [42]. Therefore, our study also linked genes with diseases by utilizing the CTD_human database to associate the 17 shared differential genes with neurological diseases (Figure 7A). The results indicated that the genes most closely related to neurological diseases among the selected differential genes were HMOX1, CP, IGF2, TNNT2, and LOX. The neurological diseases most strongly associated with these genes included the following six major conditions: status epilepticus, neurodegenerative diseases, Alzheimer’s disease, ataxia, hepatolenticular degeneration, and Parkinson’s disease.

Subsequently, we used the Jaccard similarity coefficient to measure the similarity between the complete differential gene data before and after treatment with the three pollutants and the gene lists obtained from the CTD database that are specifically associated with the six diseases mentioned above. The results showed that the three pollutants were more strongly associated with Parkinson’s disease, Alzheimer’s disease, and status epilepticus. Among them, BPA showed more significant correlation with hepatolenticular degeneration, which is a genetic disorder whose pathogenesis is closely related to defects in ATP7B [43], leading to abnormal copper metabolism and resulting in a series of neurodevelopmental disorders. [44,45]. In brief, although we only conducted an association analysis between the genes of the three pollutants and the genes of six typical diseases without delving into the mechanisms by which they trigger diseases, our results provide some support for the possibility that the three pollutants may share similar mechanisms in causing neurodevelopmental toxicity (Figure 7B).

### 3.6. Deep Analysis of the BPA Dataset Shows That Its Neurotoxicity Exhibits Gender Differences

**Figure 8:**
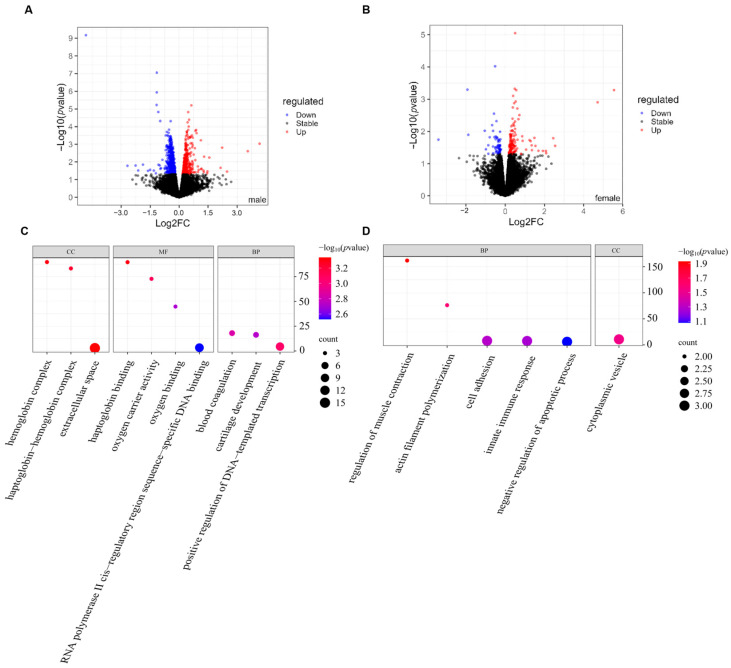
DEGs analysis of different genders in the BPA exposure group. (**A**,**B**) The volcano plot of DEGs by gender (*p* < 0.05, red indicates upregulation, and blue indicates downregulation). (**C**,**D**) The GO analysis heatmap of DEGs by gender.

BPA demonstrates significant associations with multiple neurological disorders, highlighting its notable neurotoxic potential. As an endocrine disruptor with estrogen-like activity, its toxicity appears to be gender-specific, as several studies have shown gender differences in its effects [46,47]. Therefore, we used the BPA dataset GSE249012, which includes data from both genders, to investigate the gender-specific differences in its neurodevelopmental toxicity. We found that in the male group, 85 significant DEGs were identified between the experimental and control groups (42 downregulated, 43 upregulated). In contrast, the impact of prenatal BPA exposure on females was much smaller, with only 28 genes showing significant changes (11 downregulated and 17 upregulated) (Figure 8A,B). This suggests that males may be more susceptible to the effects of BPA exposure.

The GO enrichment analysis of DEGs suggests that the relevant genes or proteins in male mice may play an important role in the extracellular space, participating in interactions between cells and the external environment as well as signal transduction. Enrichment in hemoglobin complex, globin binding, and hemoglobin-globin complex indicates that the subjects are related to oxygen transport and the process of clearing oxidized hemoglobin, which may play a role in regulating oxygen supply and maintaining blood function. The positive regulation of DNA template transcription and RNA polymerase II cis-regulatory region binding suggests that the relevant molecules may play a key role in gene expression regulation, particularly in transcriptional activation [48]. Enrichment in blood coagulation and cartilage development implies that the subjects may be involved in hemostasis and skeletal development. Overall, these enriched pathways reveal the potentially significant impact of BPA treatment on various biological processes such as immune regulation, metabolic balance, tissue repair, and gene expression regulation (Figure 8C). For female mice, the GO enrichment analysis shows that the relevant genes may participate in changes in cell morphology, immune response regulation, the control of cell death, and the maintenance of tissue structure. Specifically, muscle contraction regulation and the activation of myofiber aggregation point to dynamic changes in the cytoskeleton, which are crucial for muscle function, movement ability, and cell migration [49]. Enrichment in cytoplasmic vesicles suggests the transport of intracellular materials, signal transduction, and interactions between organelles [50]. Regulation of cell adhesion reflects cell-cell interactions, affecting tissue structure formation and homeostasis [51]. The innate immune response indicates the potential role of this gene set in immune defense, especially in rapid responses during early-stage infections. The negative regulation of apoptosis suggests that this gene set may play an important role in cell survival and tissue repair, preventing unnecessary cell death and maintaining the stability and adaptability of the organism [52,53]. In summary, these enriched pathways reveal a complex regulatory network involving the coordination of intracellular and extracellular environments, reflecting the organism’s integrated response to internal and external stimuli (Figure 8D).

**Figure 9:**
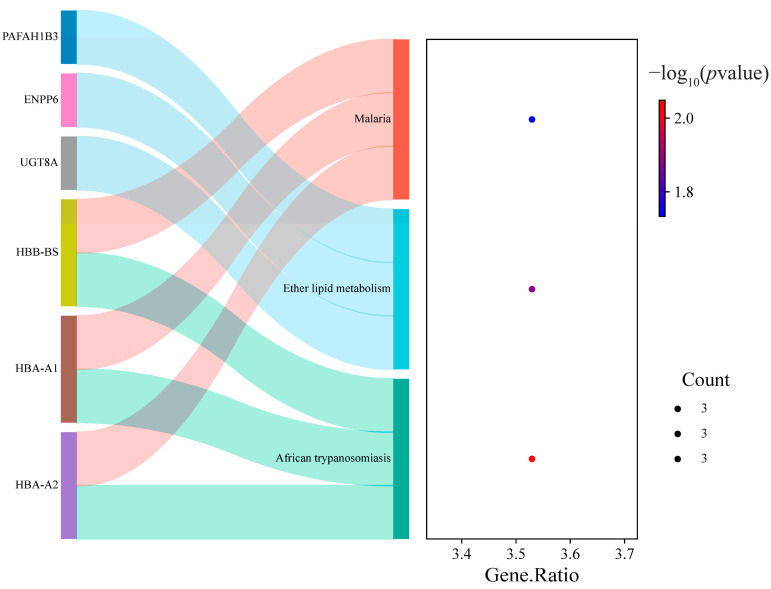
The Sankey plot of the association between male DEGs and KEGG pathways (*p* < 0.05). The color variation of the circles indicates the significance of enrichment, and the size of the circles represents the gene count.

The KEGG enrichment analysis indicates that (Figure 9), after BPA treatment, the DEGs in males are enriched in lipid metabolism, immune response, and related energy metabolism. In particular, the enrichment in ether lipid metabolism suggests that BPA may regulate the composition of cell membrane lipids, interfering with cell structure and function, especially in terms of cell signaling, membrane stability, and energy metabolism. No significantly enriched pathways were observed for the DEGs in females. In conclusion, BPA has sex dependent effects on the neurodevelopment, potentially causing greater harm to male individuals.

## 4. Conclusions

We conducted a meta-analysis of eight high-throughput studies on three environmental contaminants (ECs), offering new insights into the functional changes associated with simultaneous exposure to multiple pollutants. Our analysis identified key genes and pathways involved in neurodevelopment regulation and revealed significant overlapping gene characteristics between BPA and PSNPs, suggesting that both may influence the structure or function of the extracellular matrix. In contrast, BDE-47 may have distinct features compared to the other two chemicals. Furthermore, our study links key genes to neurological diseases, with a particularly strong correlation between BPA and hepatolenticular degeneration, highlighting the mechanism that pollutant exposure may induce gene mutations, leading to neurodevelopmental toxicity. Further examination of the BPA dataset revealed sex-specific differences in neurotoxicity, with male individuals showing a stronger response. Considering the current limitations of the datasets, a further meta-analyses will be needed in the future to enable a direct extrapolation to human species. However, this bioinformatics-based study provides new perspectives for exploring the similarities and differences in the effects of multiple pollutants and offers valuable insights for research on pollutant exposure and its link to human neurodevelopmental disorders.

## Figures and Tables

**Table 1:** Characteristics of the datasets included in our meta-analysis.

GSE	Contaminant	Species	Routes of Exposure	Dose/bw	Gender	Experiment: Control	Data Type	PMID	Date
243,612	PSNPs	Rn	IV	*200 μg/mL*	NA	3:3	Count	38,490,422	2023.9.24
229,073	BPA	Rn	OG	5000 mg/kg	NA	2:2	FPKM	38,750,585	2023.4.6
266,401	BPA	Mm	SI	50 μg/kg/day	M	3:3	Count	39,112,449	2024.5.8
249,012	BPA	Mm	OG	40 μg/kg/day	F + M	6:6	Count	38,777,957	2024.5.29
102,849	BPA	Mm	DD-C	50 mg/kg/diet	M	4:4	Count	29,763,587	2018.6.19
19,867	BDE-47	Rn	TI	0.002, 0.2 mg/kg	F	3:3	Array	21,394,737	2010.1.13
128,431	BDE-47	Hs	IV	*1 μM *	NA	2:2	FPKM	31,326,446	2022.3.1
123,458	BDE-47	Hs	IV	*1 μM*	NA	3:3	Array	31,173,147	2019.5.1

## Data Availability

Data are contained within the article.
